# Pediatric postmortem CT angiography: validation of vascular access for PMCT angiography in stillbirths, babies and toddlers

**DOI:** 10.1007/s12024-023-00726-y

**Published:** 2023-10-09

**Authors:** G. M. Bruch, P. Hofer, L. F. Ferraz da Silva, J. R. Pires-Davidson, G. A. Bento dos Santos, F. T. Fischer

**Affiliations:** 1 Institute of Forensic Medicine, LMU, Munich, Germany; 2 Institute of Forensic Medicine, Universitätsklinikum Heidelberg, Heidelberg, Germany; 3 Faculdade de Medicina, Departamento de Patologia, Universidade de São Paulo, São Paulo, SP Brazil; 4 São Paulo Autopsy Service, University of Sao Paulo, São Paulo, SP Brazil; 5 Hospital Universitário de Brasília-Radiologia, Universidade de Brasília, Brasília, DF Brazil

**Keywords:** PMCTA, Pediatric postmortem angiography, Intraosseous access, Femoral vascular access, Umbilical vascular access

## Abstract

**Purpose:**

The use of angiography in postmortem CT angiography (PMCTA) has several advantages. In adults, femoral vascular access is well established. Due to the small and specific anatomy in fetuses and infants, the technique has to be adapted, especially regarding the vascular access. The aim of this study was to evaluate vascular access for pediatric PMCTA (pedPMCTA).

**Materials and Methods:**

Ten pedPMCTAs were performed in stillbirths, babies, and one toddler. A femoral approach by cannulation of the femoral artery and vein, an umbilical approach by cannulation of the umbilical vessels, and an intraosseous approach by an intraosseous needle were evaluated by handling and resulting imaging.

**Results:**

The insertion of a cannula with a size of 18–20 G in the femoral vessels was possible in babies. An umbilical access with peripheral venous cannulas with a size of 14–20 G was feasible in stillbirths and newborns. An intraosseous access is advisable as equal alternative to umbilical and in cases where a femoral access is not possible. The most significant problem with the vascular access is the extravasation of contrast media, but this can be reduced significantly with practice.

**Conclusion:**

When performing pedPMCTA, an umbilical vascular access is recommended if an umbilical cord with open vessels is still present. Otherwise, a bone marrow access should be preferred in the presence of an arteriovenous shunt or if only the venous system needs to be shown. If that is not the case, the femoral access with the possibility to separate venous and arterial scan should be used.

## Introduction

For more than a decade, angiography has been used in postmortem computed tomography angiography (PMCTA). It has several advantages over other postmortem imaging techniques or autopsy [1, 2]. The most obvious advantage of this method is the superior vascular imaging to detect vascular malformations, vascular anomalies, and injuries. A multicenter study by Grabherr et al. [3], using a standardized protocol [4], has shown that the diagnostic quality (accuracy, sensitivity, specificity) is particularly improved by the combination of MPMCTA (multiphase postmortem CT angiography) and autopsy to improve the overall diagnostic approach in an adult corpse.

Methods of postmortem computed tomography angiography (pedPMCTA) in pediatric cases, deceased fetuses, stillbirths, and babies as well as toddlers have been published mainly as case reports, studies with few cases, or as a model experiment in fetal pigs [5–9]. Studies with mostly fetuses undergoing postmortem angiography have long been performed, but the imaging was performed as conventional radiographs (CR) with one or two planes [10–12]. Some comparisons between different types of postmortem imaging and/or autopsy have also been published [13–15]. In contrast to adults, there is no standardized protocol for pediatric PMCTA regarding contrast media, volume, or vascular access. Therefore, a standardized approach seems necessary for more reliable and comparable pediatric postmortem diagnoses.

In adults, the femoral vascular access is well established [3, 4]. However, due to the small and specific anatomy in fetuses and infants, adaptations need to be made that focus on forensically important diagnostic aspects, so the aim of this study was to evaluate different vascular accesses for pedPMCTA.

## Material and Methods

A total of ten pediatric postmortem computed tomography angiographies (pedPMCTA) were performed (Table 1). One toddler died at 16 months of age. Four bodies of babies between 3 h and 5.5 months of age and five stillbirths between 22 and 37 weeks of gestation were examined by pedPMCTA. The maximum interval between death or fetal death and pedPMCTA was 48 h. The mean weight was 3.3 kg (min. 0.5 kg, max. 9.2 kg). The mean height was 50 cm (min. 35 cm, max. 80 cm).

The main exclusion criterion was putrefaction, especially in fetuses/stillbirths. In addition, only corpse were included in which autopsy did not have to be performed immediately after delivery of the corpse for procedural reasons.

CT was performed with a Somatom Emotion 16 Siemens® HealthCare Erlangen, Germany. Native CT (120–130 kV, 50 mA, tilt 0.0, slice 1.5 mm) was performed before angiography (120 kV, 100 mA, 50 eff.mA, tilt 0.0, slice 0.75 mm). After native CT, vascular access was established, and pedPMCTA was performed with the lipophilic contrast agent Angiofil® (Fumedica AG, Muri bei Bern, Switzerland) diluted with paraffin oil (Alogro M100, Cadium Óleos, Inamar, São Paulo-SP, Brazil).

Three different vascular access routes were tested: a femoral/inguinal approach by cannulating the femoral artery and vein, an umbilical approach by cannulating the umbilical artery and vein, and an intraosseous approach by using an intraosseous needle (EZ-IO®, Vidacare LLC, Texas, USA).

Then, if the cannula position was correct, the contrast medium mixture was injected stepwise into the vasculature to show if a complete visualization of the vascular system was possible through the specific access.

### Femoral vascular access

The left femoral artery and vein have been dissected in a fashion similar to that described for corpse of adults. A small incision is made in the left inguinal area, and the subcutaneous fat is exposed. The femoral artery and vein are identified, and the vessels are looped with a suture. A 14 gauge (G) peripheral venous catheter (PVC) (Vasofix®, B. Braun, Melsungen, Germany) is placed in the femoral artery and secured with surgical sutures.

Femoral venous access is established in the same manner using an 18 G PVC. In selected cases with very small vessels, a Seldinger technique was used to place the catheters [16, 17].

### Umbilical vascular access

After a native CT is performed, the umbilical cord is shortened to a length of 2–3 cm, and the vessels (venous and arterial) are identified. The two arteries are identified by their smaller diameter and the umbilical vein by its larger diameter. A 14 G PVC without a metal mandrin is then carefully inserted into one of the two umbilical arteries. A 20 G PVC was placed in the umbilical vein. Both catheters were secured with sutures. The remaining open artery was sutured to prevent leakage of the contrast solution.

### Bone marrow access

After native CT, bone marrow access was established using the Arrow® battery-powered bone drill and an EZ-IO® Intraosseous Vascular Access System (Vidacare LLC, Texas, USA) originally developed for emergency medicine. A 15 mm 15 G bone needle is inserted into the right side of the tibial tuberositas. The insertion for the pedPMCTA is performed according to the pediatric emergency application, where the 15 mm needle length is recommended for living patients weighing between 3 and 39 kg. The needle is inserted into the tibial tuberositas until a noticeable loss of resistance is felt, indicating penetration of the bone marrow of the right tibia. Subsequently, another native CT is performed to verify the position of the needle in the bone marrow of the right tibia. The internal drill bit was removed, and a 15 cm infusion line is connected to the remaining infusion cannula.

## Results

### Femoral vascular access

**Fig. 1:**
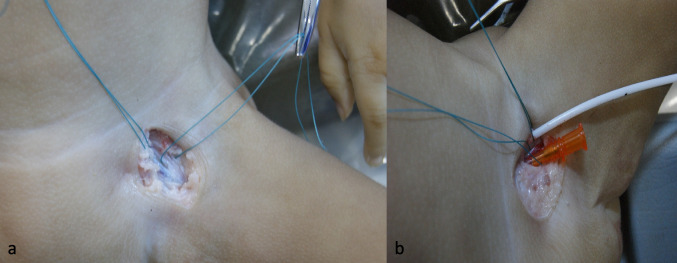
**a**, **b** Preparation and insertion of an arterial vascular access with a CVC and venous vascular access with a 20 G intravenous cannula

The body of the baby (length 80 cm, weight 4.6 kg), who died at the age of 5.5 months, was vascularized through the femoral vein (Fig. 1a, b). After venous injection of 10 ml of contrast medium mixture, the control CT showed a contrast leak at the proximal part of the left external iliac vein where the central venous catheter (CVC) was placed. Therefore, the right femoral vein was prepared in the same manner, and a new venous access was placed. Again, an 18 G intravenous cannula (Vasofix®, B. Braun, Melsungen, Germany) was used. The venous cannula was then easily inserted.

For arterial access, the femoral artery was prepared on the left side. After visualization of the artery, a 20 G intravenous cannula (Vasofix®, B. Braun, Melsungen, Germany) was inserted into the vessel, and 10 ml of the contrast agent mixture was injected. The guiding CT showed no leakage.

No leakage was detected in the subsequent test of 5 ml venous and 20 ml arterial contrast. More contrast was then gradually injected with a 10 ml syringe to obtain the clearest image of the vessels.

Another baby (length 60 cm, weight 6.0 kg) who died at the age of 10 weeks already had a central venous catheter from a previous emergency treatment. Therefore, this intravenous cannula was used to inject the contrast medium mixture in order to have at least a representation of the venous vessels.

A toddler (height 80 cm, weight 9.2 kg), 16 months of age, also had an intraosseous needle from a previous emergency treatment. The femoral artery was prepared as described, and an intravenous cannula was inserted. In this case, the contrast agent mixture was injected via the femoral artery for the arterial phase and via the intraosseous needle for the venous phase after the CT scan.

In the last two cases, the test injection was omitted, and the total amount of contrast was injected, first arterial and then, after a CT scan, venous.

### Umbilical vascular access

**Fig. 2:**
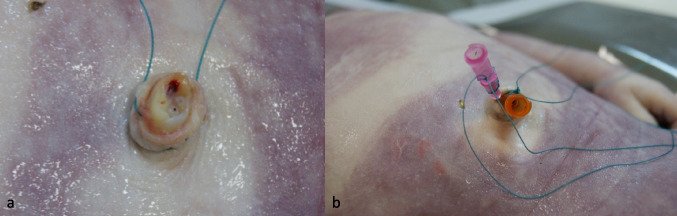
**a** Umbilical cord after shortening it and identifying the vessels. **b** Umbilical cord after insertion of the arterial and venous cannula

Venous and arterial umbilical vascular access was performed in a total of four stillbirths and one deceased neonate (Table 1). The newborn was 3 h old at the time of death (body length 40 cm, body weight 3.11 kg). The stillbirths (body length 30–45 cm, body weight 0.48–2.50 kg) were born between 22 and 37 weeks of gestation. In all cases, it was possible to position the cannulas at the umbilical vessels (Fig. 2a, b).

**Table 1:** Overview of the scanned bodies, their vascular access, and materials employed (age in months (mon), weeks (w), and hours (h) or gestation age (GA) in weeks (w))

	Age	Weight (kg)	Height (cm)	Vascular access	Material employed
Baby	5.5 mon	4.50	80	Femoral vessels	CVC, peripheral intravenous cannula
Stillbirth	GA 37 w	2.50	40	Umbilical vessels	Peripheral intravenous cannula
Newborn	3 h	3.11	40	Umbilical vessels	Peripheral intravenous cannula
Stillbirth	GA 30 w	1.57	45	Umbilical vessels	Peripheral intravenous cannula
Stillbirth	GA 22 w	0.48	35	Umbilical vessels	Peripheral intravenous cannula
Stillbirth	GA 22 w	1.20	30	Umbilical vessels	Peripheral intravenous cannula
Baby	2 mon	2.50	50	Tibia bone marrow	Bone Drill EZ-IO®
Stillbirth	GA 23 w	1.41	40	Tibia bone marrow	Bone Drill EZ-IO®
Baby	10 w	6.00	60	Femoral vein	CVC, peripheral intravenous cannula
Toddler	16 mon	9.20	80	Tibia bone marrow and femoral artery	Bone Drill EZ-IO® (venous) and intravenous cannula (arterial)
*n* = 10	Mean:	3.30 kg	50 cm

**Fig. 3:**
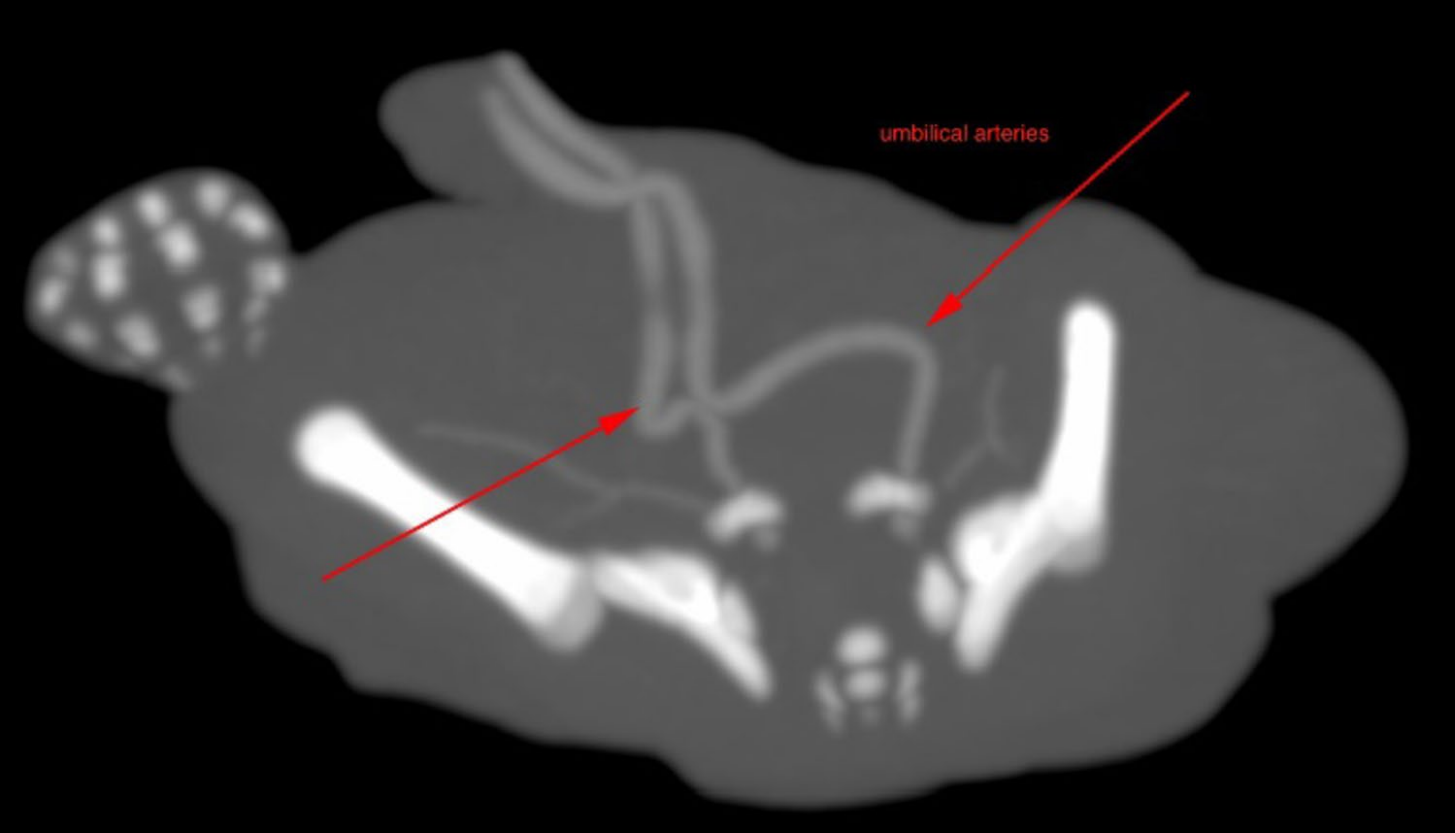
Imaging after injecting 1 ml contrast agent mix into each of the umbilical arteries to test side-related differences

**Fig. 4:**
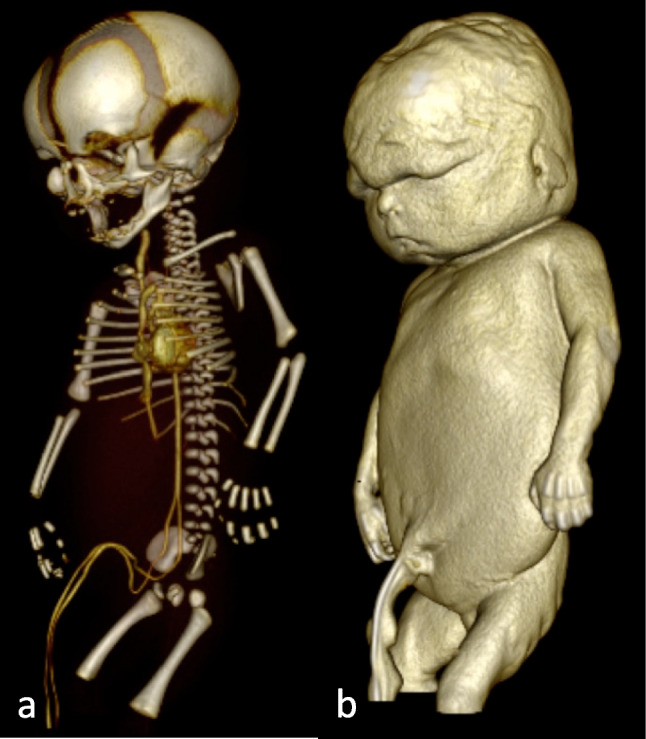
Fetus after injection of two milliliter contrast agent via the arterial umbilical vascular access in a 3D reconstruction. **a** The arterial vascular system can be seen. **b** The confluence of the umbilical vessels

After a test injection of 1–2 ml of contrast medium mixture (Fig. 3), PMCTA could be performed in all cases. In only one case, contrast leaked from the arterial vessel directly at the entry of the umbilical vessels, causing extravasation into the peritoneal cavity. In this case, an intravenous cannula (20 G) was later inserted into the second umbilical artery, and after a successful test of 2 ml of contrast mixture, postmortem angiography could be performed in stages (Fig. 4a, b).

### Bone marrow access

**Fig. 5:**
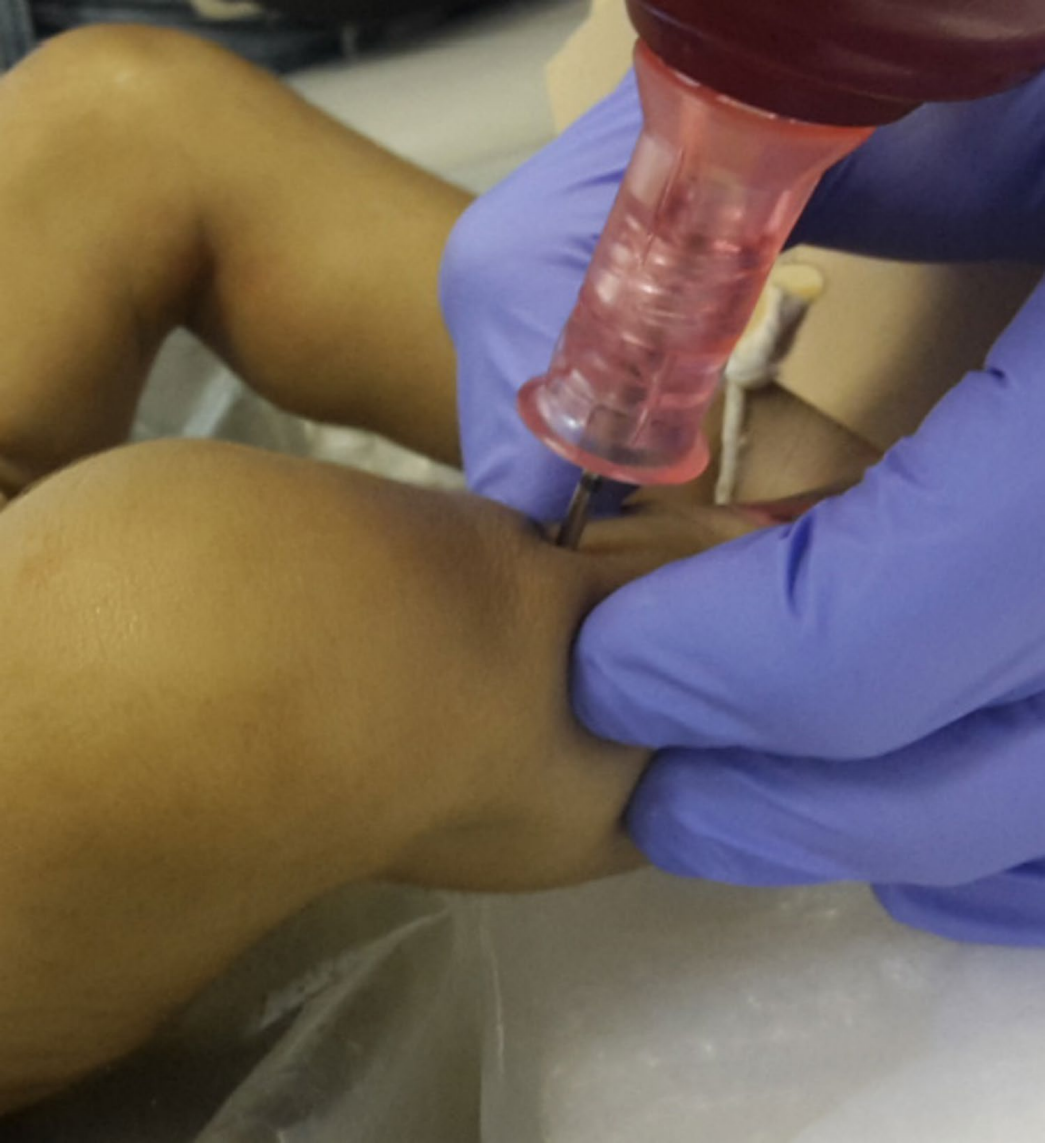
Drilling a bone marrow access into the right tibia

**Fig. 6:**
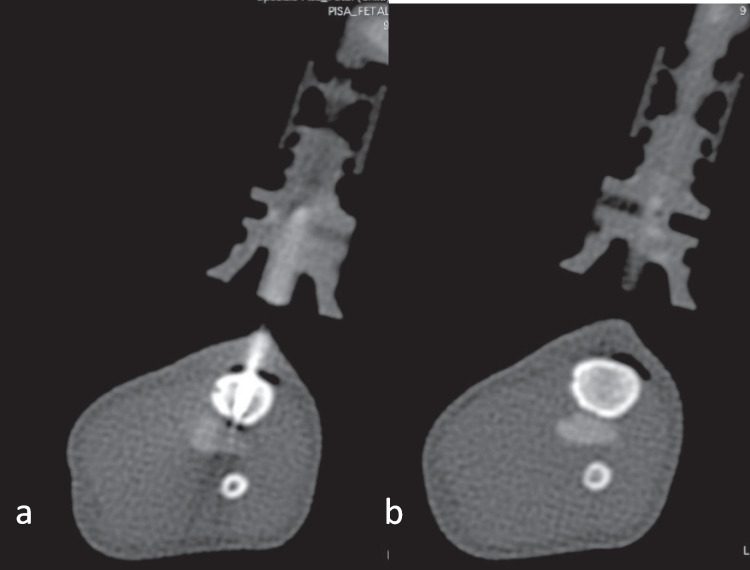
**a**, **b** Bone marrow access with leakage in the muscle

Bone marrow access was performed as described on the body of a toddler (16 months, weight 9.2 kg, length 80 cm), a baby who died at the age of two months (50 cm, 2.5 kg, Fig. 5), and a stillborn baby (GA 23rd week, 40 cm, 1.4 kg). Another CT scan was performed after injection of 2 ml of contrast. In the stillbirth, the anterior and posterior corticalis of the tibia were punctured due to its small diameter, resulting in contrast leakage into the surrounding tissues (Fig. 6a, b). In this case, the latter intraosseous contrast administration was stopped, and an umbilical vascular access was prepared, followed by a successful pedPMCTA.

**Fig. 7:**
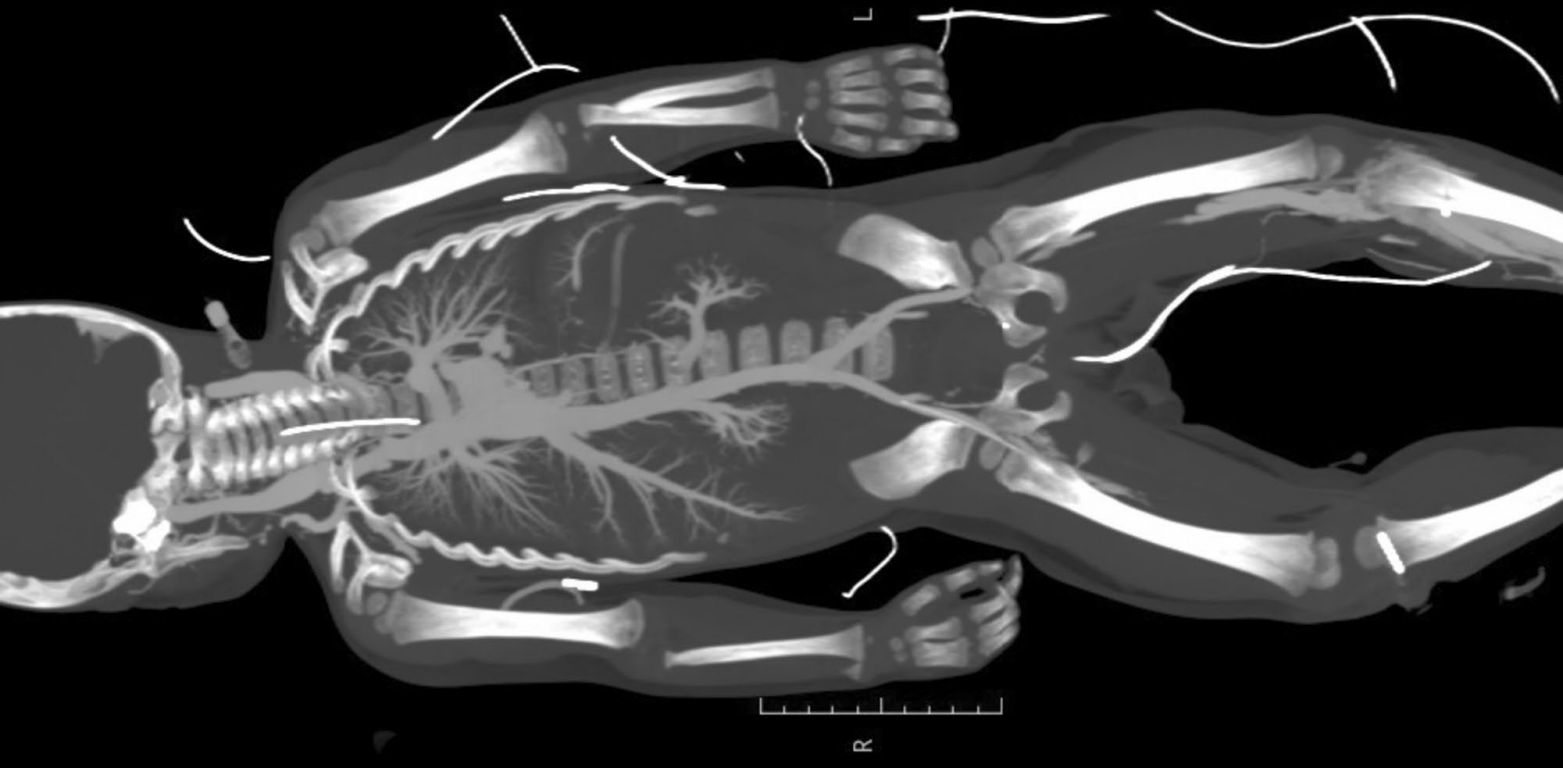
Venous vascular system after injecting 220 ml contrast mixture via a tibial bone marrow access

In the toddler who already presented with a tibial bone marrow needle, this device was used to successfully perform a venous phase in the procedure (Fig. 7).

In the case of the deceased neonate, vascular access testing showed no leakage, allowing a continuous distribution of the contrast medium. The contrast was first distributed from the right lower leg into the venous system and, with the Botalli ductus still open, into the arterial system. Thus, a pedPMCTA was successfully performed with opacification of the entire vasculature.

## Discussion

A total of ten pediatric postmortem computed tomography angiographies were performed for this evaluation. The cases included four age groups: stillbirth, perinatal, baby, and toddler.

The fetal vascular anatomy usually shows a physiologically open ductus arteriosus also Ductus Botalli) and/or patent foramen ovale with no separation between arterial and venous circulation. Therefore, arterial and venous catheters for pedPMCTA do not regularly provide separate images of the arterial or venous system. The decision of which approach is most useful should be based on the primary diagnostic focus of the case.

Vascular access via the umbilical vein and artery for pedPMCTA has been described in the literature [6, 11, 18, 19]. It is an easy way to perform angiography in fetuses, neonates, and/or stillbirths. The insertion of an intravenous cannula into the umbilical cord is easy and the corresponding vessels can be easily identified in most cases. An arterial and venous phase can be performed minimally invasively, without skin incision. The disadvantage is that there may be extravasation of contrast medium into the peritoneal cavity. Depending on the amount of contrast injected, this can make evaluation in the abdominal cavity extremely difficult, if not impossible. As a result, relevant findings may be lost at this site. In addition, vascular access via the umbilical cord is limited to fetuses, stillbirths, or babies who died shortly after birth.

Femoral vascular access has been performed in babies and children [5]. It has been tested and established many times, especially in adults in the context of PMCTA [3, 4, 20]. The advantage is that the femoral vein/artery in the right or left inguinal region can be easily accessed and prepared in a known anatomical position. Bilateral preparation, e.g., in case of leakage, is possible. In addition, this vascular access can be performed on any corpse. Furthermore, many physicians performing PMCTAs are already experienced in preparing these vessels. However, it should be kept in mind that especially in stillborn and deceased babies, the inguinal vessels are still very small due to their age. Establishing vascular access is therefore difficult due to the small diameter. In addition, damage to the inguinal vessels carries the risk of leakage of the contrast medium into the lower abdomen. This can make it difficult to assess abnormalities or defects at the site. In addition, a skin incision must be made for each femoral vascular access. If an autopsy is performed after pedPMCTA, this incision is usually incorporated into the autoptic skin incision. However, if no autopsy is ordered, the sutured skin incision remains on the corpse as a remnant of the angiography. However, it is still a minimally invasive procedure. To the best of our knowledge, an osseous vascular access for postmortem angiography has not been reported in the literature, but it has long been established as a vascular access in emergency medicine [21–23]. According to a known case report, intraosseous vascular access for contrast injection in pediatric CT angiography is possible in an emergency setting and produces high-quality images with the use of conventional contrast agents [24]. Establishing vascular access with this method is simple and fast, because after finding the correct anatomical position, only the bone needle needs to be drilled with an appropriate drill device. Another advantage is that several optional sites are available for bone marrow access, such as humerus, tibia and calcaneus. Furthermore, in the event of a leak, only a local collection of contrast medium remains at the insertion site. This does not lead to an overlapping of possible findings in the abdominal cavity. In addition, only a small skin puncture remains on the corpse after pedPMCTA. Venous access can only be used to fill an arterial phase if the baby has an open patent ductus arteriosus, foramen ovale, or another arteriovenous shunt, such as an atrial septal defect (ASD) or ventricular septal defect (VSD). Intraosseous vascular access may also be chosen if there is a specific (forensic) question where imaging of the venous vasculature is sufficient or even preferable. This may be the case when imaging of the bridging veins (suspected abusive head trauma) or the sagittal sinus (suspected sinus venous thrombosis) is required. In our experience, bone access is usually not possible in very small corpses, as the smallest available bone needle is recommended for weights of three kilograms or more. In our test, this needle was easily inserted in a 2.5 kg corpse. A CT-controlled insertion is also possible. In addition, the appropriate equipment must be purchased for this vascular access, as a bone drill is required. The use of oil did not cause any problems in this approach. In all cases, evaluation of the vascular system was possible after postmortem angiography, with very good contrast between the arterial and venous structures.

## Conclusion

**Fig. 8:**
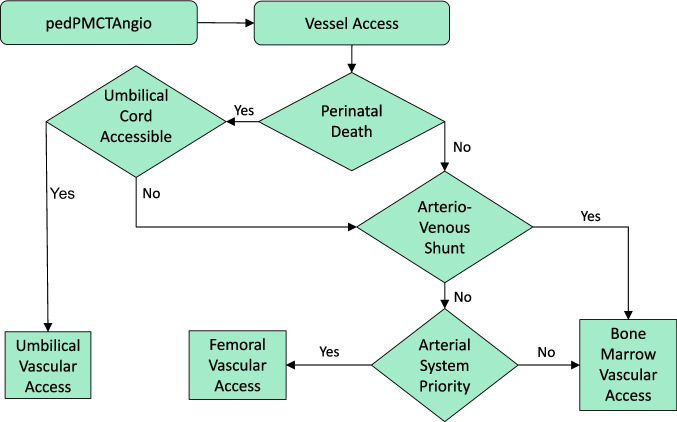
Decision algorithm for the most suitable vascular access

When performing pedPMCTA in fetuses, stillbirths, or perinatal patients, umbilical vascular access is recommended if an umbilical cord with open vessels is still present. If this is not the case, bone marrow access should be preferred for ease of use in the presence of a physiologic or pathologic arteriovenous shunt or when only the venous system needs to be visualized. If this is not the case, the femoral vessels can be dissected (Fig. 8). In addition, the use of 18–20 G arterial and 14 G venous intravenous cannula for both umbilical and femoral vascular access has proven to be simple, cost-effective, and practical. Intraosseous access appears to be very promising due to its ease of use. The main problem with vascular access is extravasation of contrast outside the vessels, but this has been significantly reduced with practice. Practice seems to be necessary. It seems useful to perform the studies in specialized centers. Further studies are needed to evaluate its applicability and advantages in abusive trauma cases, where a minimally invasive approach seems to be advantageous.

## Data Availability

N/A.
